# Mechanical testing of a device for subcutaneous internal anterior pelvic ring fixation versus external pelvic ring fixation

**DOI:** 10.1186/1471-2474-15-111

**Published:** 2014-03-31

**Authors:** Georg Osterhoff, Simon Tiziani, Stephen J Ferguson, Gregor Spreiter, Max J Scheyerer, Gian-Leza Spinas, Guido A Wanner, Hans-Peter Simmen, Clément ML Werner

**Affiliations:** 1 Division of Trauma Surgery, University of Zurich, Raemistrasse 100, Zurich 8091, Switzerland; 2 Institute for Biomechanics, ETH Zurich, HCI-E355.2 Wolfgang-Pauli-Strasse 10, Zurich 8093, Switzerland

**Keywords:** Pelvic fracture, Pelvic ring injury, Internal fixation, External fixation, Subcutaneous internal anterior fixation

## Abstract

**Background:**

Although useful in the emergency treatment of pelvic ring injuries, external fixation is associated with pin tract infections, the patient’s limited mobility and a restricted surgical accessibility to the lower abdomen. In this study, the mechanical stability of a subcutaneous internal anterior fixation (SIAF) system is investigated.

**Methods:**

A standard external fixation and a SIAF system were tested on pairs of Polyoxymethylene testing cylinders using a universal testing machine. Each specimen was subjected to a total of 2000 consecutive cyclic loadings at 1 Hz with sinusoidal lateral compression/distraction (+/−50 N) and torque (+/− 0.5 Nm) loading alternating every 200 cycles. Translational and rotational stiffness were determined at 100, 300, 500, 700 and 900 cycles.

**Results:**

There was no significant difference in translational stiffness between the SIAF and the standard external fixation when compared at 500 (p = .089), 700 (p = .081), and 900 (p = .266) cycles. Rotational stiffness observed for the SIAF was about 50 percent higher than the standard external fixation at 300 (p = .005), 500 (p = .020), and 900 (p = .005) cycles. No loosening or failure of the rod-pin/rod-screw interfaces was seen.

**Conclusions:**

In comparison with the standard external fixation system, the tested device for subcutaneous internal anterior fixation (SIAF) *in vitro* has similar translational and superior rotational stiffness.

## Background

External fixation has been an established technique in the emergency treatment of pelvic ring injuries [1,2]. With external fixation, reduction of the inner pelvic diameter can be achieved within minutes, thereby limiting venous haemorrhage into the lesser pelvis [3]. Besides this direct effect on the extent of blood loss in the acute situation, external fixation reduces motion between the pelvic fragments and thus helps to maintain haemostasis during the first days after the trauma [4,5].

It has been shown that supra-acetabular positioning of the pins leads to improved anchorage of the pins and a higher construct stability when compared to a pin placement into the iliac crest [6].

Particularly for pelvic injuries with posterior instability, appropriate stability can only be achieved by combined anterior and posterior fixation or a sufficient posterior fixation alone [7,8].

Thus, definitive internal pelvic stabilization is usually applied as soon as the patient’s condition allows for more extensive surgery [9].

Besides usually serving only as a temporary stabilization, the use of external fixation of pelvic fractures is associated with further disadvantages. Pin tract infections are common and can occur in up to 50% of cases [10]. Rolling the patient side-to-side, sitting and lying in prone position is limited. Especially in patients with concomitant intra-abdominal injuries, where multiple revisions might be necessary, an anterior frame impairs the access to the lower abdomen. In addition, achieving stability by external fixation in severely obese patients with a large distance between the pelvis and the connecting rods is almost impossible, due to abundant abdominal soft tissue.

Therefore, a subcutaneous internal anterior screw-rod fixation has been suggested that can be applied within a comparable time to an external fixateur, while avoiding the disadvantages of the external implant [11]. This new technique has already been tested in several case series, where indeed very low rates of wound infections were observed when compared to those of external fixation. Yet, it was associated new complications like irritation of the lateral femoral cutaneous nerve or heterotopic ossifications [11-13].

With the development of minimal-invasive spinal instrumentation systems, there are new possibilities to perform this technique of subcutaneous internal anterior fixation (SIAF) in a less invasive fashion.

Little is known about the mechanical properties of such instrumentation. Failure to achieve stable fixation can result in the detachment of blood clots at the fracture site and therefore put the patient at risk for recurrent bleeding. As well, loosening of the rod-screw interface would require revision surgery.

The purpose of this study was to investigate the *in vitro* mechanical stability of a device for subcutaneous internal anterior fixation and compare it to a standard external fixation.

## Methods

### Testing samples

The experimental setup was designed to progressively measure the fatigue life or damage accumulation of the rod-pin/rod-screw interfaces. Two fixation techniques were tested on pairs of Polyoxymethylene testing cylinders with a diameter of 70 mm (*Delrin*, DuPont, Wilmington, DE, USA). These cylinders provided sound purchase of the pelvic pins and screws thereby simulating optimum anchorage in the pelvic bone.

**Figure 1:**
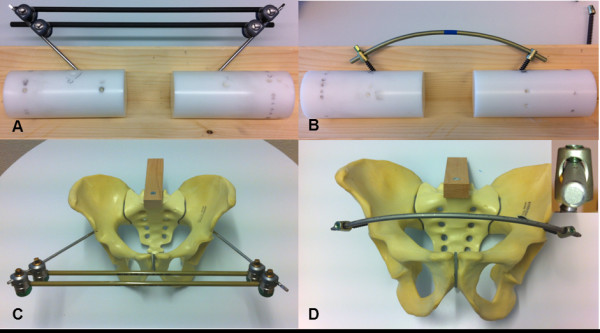
**Testing samples.** Reproducing angles and distances measured on similar pelvic models **(C, D)**, a standard external fixateur **(A)** and a subcutaneous internal anterior fixation system **(B)** were mounted on pairs of Polyoxymethylene testing cylinders. The small image on the right shows a close-up of the coupling of the subcutaneous internal anterior fixation system.

In the group EXTERNAL (n = 3), a standard external fixateur (Hoffmann II, Stryker, Kalamazoo, MI, USA) was mounted (Figure 1A) using two apex self-drilling pins (diameter 5 mm) each anchored in one of on two separate testing cylinders. Angles and distances between the two pins were chosen in a way to reproduce the conditions as measured for the supra-acetabular channel on a pelvic model (*Pelvis*, Synbone, Malans, Switzerland) (Figure 1C). Two connecting carbon fiber rods (diameter 8 mm) were attached using pin-to-rod couplings according to the manufacturer’s instructions. Like in clinical routine, we aimed for torque forces exceeding that of a torque-limiter. Thus, all couplings were tightened with maximum effort by the same person (S. T.).

In the group SIAF (n = 3), two 8-mm-screws (*Iliac Multiaxial Screw*, Legacy, Medtronic, Minneapolis, MN, USA) were placed in two testing cylinders in exactly the same way as described for the supra-acetabular pins in the EXTERNAL group Figure 1B, D). The screws were connected by a contoured titanium rod (diameter 8.5; Longitude, Medtronic, Minneapolis, MN, USA). Couplings in the polyaxial screw heads were tightened by the same person (S. T.) with a torque screwdriver according to the manufacturer’s instructions (Legacy, Medtronic, Minneapolis, MN, USA).

### Mechanical testing

In patients, two basic loading scenarios are likely after provisional stabilization of pelvic ring fractures. First, rolling the patient side-to-side is common (e.g. when changing bed-linen) which would result in lateral compression forces on the pelvic fixation construct in the transversal plane. Second, bending of the hip or transfer to a chair would result in torque forces in the sagittal plane.

**Figure 2:**
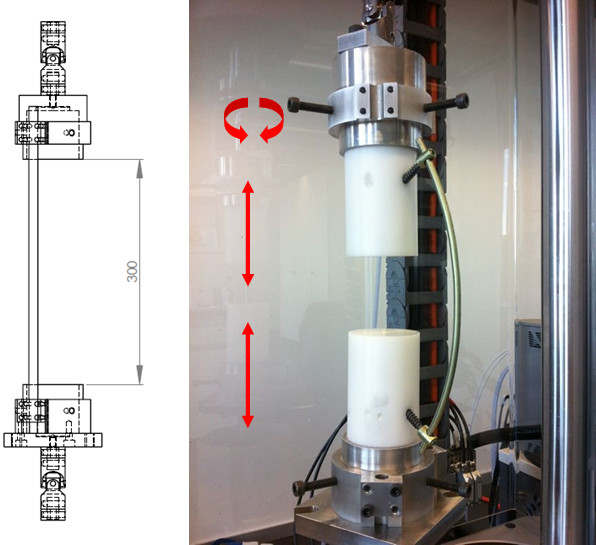
**Experimental setup.** On a universal testing machine, 2000 consecutive cyclic loadings were applied with sinusoidal lateral compression/distraction and torque loading alternating every 200 cycles. The cylinder axes of the samples were aligned with the axis of the actuator using universal joints to eliminate confounding bending moments (see dimensional sketch on the left).

Thus both fixation constructs were tested by alternating cycles of lateral compression/distraction (i.e. following the cylinder axis) and torque forces (i.e. counter-rotation of the cylinders about the central axis) using a universal testing machine (ElectroPulse E10000, Instron, High Wycombe, UK). The cylinder axes of the samples were aligned with the axis of the actuator using universal joints to eliminate confounding bending moments (Figure 2). After observing a displacement of clinical relevance (> 25 mm) at +/−50 N, this load was chosen for the experimental setup.

Each specimen was subjected to total of 2000 consecutive cyclic loadings at 1 Hz with sinusoidal lateral compression/distraction (+/−50 N) and torque (+/− 0.5 Nm). One thousand cycles of rotation and 1000 cycles of compression/distraction with the mode of loading alternating every 200 cycles were applied.

During testing, loading forces and displacement were registered by a +/−1000 N (compression/distraction forces) and a 25 Nm (torque forces) load cell and translational and rotational stiffness were calculated. Failure resulting in immediate stop of testing included: displacement beyond the machine’s range of motion (+/− 30 mm in the transversal plane, +/− 135 degrees rotation) or interface failure.

The following parameters were evaluated:

### Translational stiffness [N/mm]

A measure of the extent to which the fixation system resisted deformation in response to a force simulating lateral compression of the pelvis. This value was determined at 100, 300, 500, 700 and 900 cycles.

### Rotational stiffness [Nm/degree]

A measure of the extent to which the fixation system resisted deformation in response to a moment simulating torque of the pelvis. This value was determined at 100, 300, 500, 700 and 900 cycles.

### Statistical analysis

After preliminary testing, a sample size calculation was performed using PS Power and Sample Size Calculations 3.0 (alpha error: 0.05) [14].

With an expected difference in means of 0.5 N/mm and a standard deviation of 0.15 N/mm for translational stiffness and an expected difference in means of 0.08 Nm/degree and a standard deviation of 0.025 Nm/degree for rotational stiffness the calculated number of samples to be able to reject the null hypothesis that the population means of the experimental and control groups are equal with a probability (power) of 0.8 was 3 per group.

After testing for normal distribution by a Shapiro-Wilk test, comparison of translational and rotational stiffness was done using a paired T-Test with SPSS for Windows V14.0 (SPSS, Chicago, Illinois, USA). Differences were considered significant for values of *p* < 0.05.

## Results

### Translational stiffness

**Figure 3:**
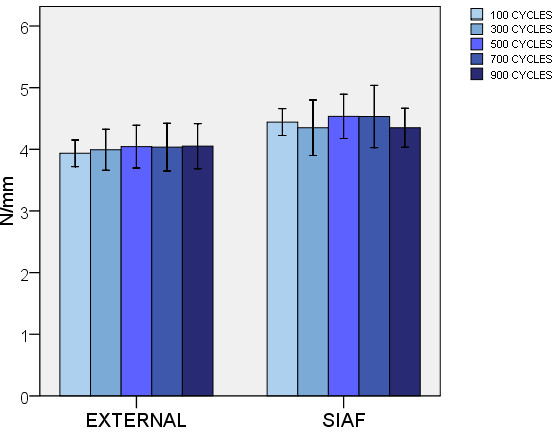
**Translational stiffness.** EXTERNAL: standard external fixation. SIAF: subcutaneous internal anterior fixation. Error bars represent 2 standard deviations.

There was a slight but not significant difference, when tested in response to a force simulating lateral compression of the pelvis, between the stiffness observed for the internal anterior fixation (SIAF) and the standard external fixation (EXTERNAL) when compared at 500 (p = .089), 700 (p = .081), and 900 (p = .266) cycles. At 100 (p = .038) and 300 (p = .021) cycles, however, the translational stiffness was even slightly higher in the SIAF group (Figure 3).

### Rotational stiffness

**Figure 4:**
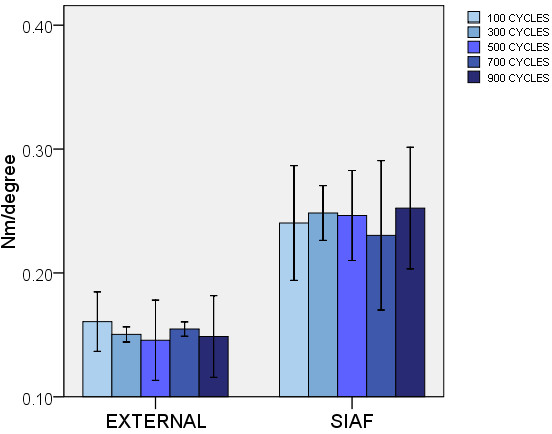
**Rotational stiffness.** EXTERNAL: standard external fixation. SIAF: subcutaneous internal anterior fixation. Error bars represent 2 standard deviations.

When tested in response to a force simulating torque of the pelvis, the stiffness observed for the subcutaneous internal anterior fixation (SIAF) was about 50 percent higher than the standard external fixation (EXTERNAL) at all times of measurement. This was statistically significant when compared at 300 (p = .005), 500 (p = .020), and 900 (p = .005) cycles. At 100 (p = .050) and 700 (p = .050) cycles, the level of significance was not achieved (Figure 4).

Temporary deformation was solely caused by elastic bending of the rods, screws and pins, the rod-pin/rod-screw interfaces did not show any loosening or failure with the applied loads. Persistent plastic deformation was not seen.

## Discussion

Supra-acetabular external fixation of pelvic ring injuries is an efficient tool to minimize blood loss in the acute situation and to maintain haemostasis during the first days after the trauma [1-6]. However, pin tract infections, the patients’ limited mobility and a restricted surgical accessibility to the lower abdomen are noticeable disadvantages of this technique. In 2009, Kuttner et al. introduced the idea of a subcutaneous internal anterior fixation of the pelvis [11].

This study tested the *in vitro* mechanical stability of a subcutaneous internal anterior fixation system (SIAF) in comparison to a standard external fixation (EXTERNAL). We used an experimental setup with a focus on the rod-pin/rod-screw interface, which was thought to be the weakest link within both fixation techniques and simulated forces resulting from side-to-side rolling of a patient (translational stiffness) and forces resulting from bending of the hip (rotational stiffness). The SIAF system showed a similar to slightly higher translational stiffness than the EXTERNAL system and a significantly higher rotational stiffness at up to 900 loading cycles. It is important to note that this combined implant-construction stiffness is a function of rod- and pin/screw stiffness, pin/screw-rod interface motility and the swinging distance of the implant over the bone surrogate. It is our conviction that this difference is largely caused by the large swinging distance of the pins over the bone in external fixation. This problem is partly eliminated by the positioning of the rod in SIAF.

Several case series using a subcutaneous internal anterior fixation showed good short-term results with only few complications related to implant failure [11-13]. Our *in vitro* data is consistent with these clinical experiences. In addition, there is one recent mechanical study comparing a similar subcutaneous anterior fixation technique with anterior external and plate fixation suggesting superior stiffness of the SIAF-like system [15]. The latter study, however, uses only one single connecting rod with the external fixation, which is known to have inferior mechanical stability [16] and therefore usually does not represent the clinical routine. In contrast to our study, a cyclic loading setup was not used by Vigdorchik et al. [15]. In most *in vivo* cases, however, postoperative fragment dislocation is not a result of a single trauma or force, but rather occurs due to repetitive motion (e.g. by nursing bedside assistance or physiotherapy) as is suggested by the typical appearance of symmetric aseptic loosening of the pins in many cases [10]. Therefore, cyclic loading seems to better approximate clinical loading scenarios.

To date, there exists no standard in mechanical testing of pelvic ring fixations. Generally, a cadaver or synthetic pelvis is tested with large variations in force vectors’ direction, quantity and point of application [4,15,17-21]. Most experimental setups ignore forces resulting by acceleration when walking and ignore force vector realignment by abductor and adductor muscles [17,22,23].

There is also no gold standard for external fixation configurations in the treatment of pelvic ring injuries, even single-rod fixation has been described [15]. A double-rod configuration as used in our study, however, has been considered to provide sufficient stability [16].

The stability of pelvic fixation systems is defined by their stiffness and by possible failure of the rod-pin/rod-screw interfaces. As data for *in vivo* loading of the pelvic ring are limited, the test was designed to progressively measure the fatigue life or damage accumulation of these rod-pin/rod-screw interfaces [24]. A simplified setup was chosen with a focus on the stiffness of the implants themselves as well as on the specified interfaces. By choosing this test protocol, confounding factors were avoided, such as the quality of screw/pin anchorage, which depends on bone quality and surgical application. With the results of this study, no conclusions can be drawn on the implant’s behaviour at the implant-bone-interface. It is possible that the increased stiffness of the SIAF system prevents loosening of bone anchorage, or also that it even promotes loosening by increased stresses at the implant-bone-interface [25]. The very low implant loosening rates in the few known clinical series using the SIAF [11-13] support the idea of improved anchorage – due to a higher stiffness and stability and due to the greater diameter of the SIAF screws.

All the latter clinical studies applied the SIAF in combination with posterior fixation. Future studies may further analyse the implant-bone-interface by the use of cadaver testings or finite element models [26].

Our *in vitro* data suggest the possibility of even an isolated use of the SIAF for fractures where external fixation alone would be considered adequate.

This would provide a quick intervention with early and simplified mobilization of the patient. It is known that, in the treatment of pelvic ring injuries, early mobilization significantly reduces posttraumatic complications and morbidity [5]. In fractures with intact posterior sacroiliac and sacro-tuberous ligaments (like LC II and APC II), posterior stabilization has to be postponed in some cases. By providing higher stability and lower infection and loosening rates [11-13], isolated preliminary anterior fixation with SIAF might be an alternative to external fixation. However, the promising results of this study have yet to be proven *in vivo*.

In this context, it is important to mention that SIAF seems to be associated with irritation of the lateral femoral cutaneous nerve in up to 27% of cases and with heterotopic ossifications in up to 32% [11-13]. In addition, components of SIAF are close to important anatomic structures, like the femoral vascular bundle and the urinary bladder [27].

Hence, careful adherence to the surgical anatomy is crucial.

## Conclusions

In comparison with the standard external fixation system, the tested device for subcutaneous internal anterior fixation (SIAF) *in vitro* has similar translational and superior rotational stiffness. This technique might provide a useful tool in the treatment of pelvic ring injuries, especially in case of patients with concomitant abdominal injuries or risk factors for pin tract infection.

## Competing interests

The authors declare that they have no competing interests.

## Authors’ contributions

GO, ST, SF, and CW participated in the study’s design and planning. GO, ST, SF, GS, G-LS, and CW carried out the testing. GO, SF, and MS performed the data analysis. GO, SF, GW, H-PS, and CW drafted/revised the final manuscript. All authors read and approved the final manuscript.

## Pre-publication history

The pre-publication history for this paper can be accessed here:

http://www.biomedcentral.com/1471-2474/15/111/prepub
